# Enhancing Proton Radiosensitivity of Chondrosarcoma Using Nanoparticle-Based Drug Delivery Approaches: A Comparative Study of High- and Low-Energy Protons

**DOI:** 10.3390/ijms252111481

**Published:** 2024-10-25

**Authors:** Mihaela Tudor, Roxana Cristina Popescu, Ionela N. Irimescu, Ann Rzyanina, Nicolae Tarba, Anca Dinischiotu, Liviu Craciun, Tiberiu Relu Esanu, Eugeniu Vasile, Andrei Theodor Hotnog, Mihai Radu, Gennady Mytsin, Mona Mihailescu, Diana Iulia Savu

**Affiliations:** 1 Department of Life and Environmental Physics, Horia Hulubei National Institute of Physics and Nuclear Engineering, Reactorului 30, P.O. Box MG-6, 077125 Magurele, Romania; mihaela.tudotr@nipne.ro (M.T.); roxana.popescu@nipne.ro (R.C.P.); mradu@nipne.ro (M.R.); 2 Faculty of Biology, University of Bucharest, Splaiul Independentei 91-95, 050095 Bucharest, Romania; anca.dinischiotu@bio.unibuc.ro; 3 Department of Bioengineering and Biotechnology, Faculty of Medical Engineering, National University for Science and Technology Politehnica of Bucharest, Gheorghe Polizu Street, 1-7, 011061 Bucharest, Romania; 4 Applied Sciences Doctoral School, National University for Science and Technology Politehnica of Bucharest, 060042 Bucharest, Romania; ionela.irimescu@stud.fsa.upb.ro; 5 Laboratory of Nuclear Problems, Joint Institute for Nuclear Research, 6 Joliot-Curie Street, 141980 Dubna, Moscow Region, Russia; rzjanina@mail.ru (A.R.); mytsin@mail.ru (G.M.); 6 Doctoral School of Computer Sciences, National University for Science and Technology Politehnica of Bucharest, 060042 Bucharest, Romania; nicolae.tarba@upb.ro; 7 Radiopharmaceutical Research Centre, Horia Hulubei National Institute of Physics and Nuclear Engineering, 077125 Magurele, Romania; cliviu@nipne.ro (L.C.); tiberiu.esanu@nipne.ro (T.R.E.); 8 Faculty of Applied Physics, National University for Science and Technology Politehnica of Bucharest, 060042 Bucharest, Romania; eugeniu.vasile@upb.ro; 9 Applied Nuclear Physics Department, Horia Hulubei National Institute of Physics and Nuclear Engineering, Reactorului 30, P.O. Box MG-6, 077125 Magurele, Romania; andrei.hotnog@nipne.ro; 10 Holographic Imaging and Processing Laboratory, Physics Department, National University for Science and Technology Politehnica of Bucharest, 060042 Bucharest, Romania; mona.mihailescu@upb.ro; 11 Centre for Research in Fundamental Sciences Applied in Engineering, National University for Science and Technology Politehnica of Bucharest, 060042 Bucharest, Romania

**Keywords:** chondrosarcoma, iron oxide nanoparticles, drug delivery, doxorubicin, protons, radiosensitization, hyperspectral imaging

## Abstract

To overcome chondrosarcoma’s (CHS) high chemo- and radioresistance, we used polyethylene glycol-encapsulated iron oxide nanoparticles (IONPs) for the controlled delivery of the chemotherapeutic doxorubicin (IONP_DOX_) to amplify the cytotoxicity of proton radiation therapy. Human 2D CHS SW1353 cells were treated with protons (linear energy transfer (LET): 1.6 and 12.6 keV/µm) with and without IONP_DOX_. Cell survival was assayed using a clonogenic test, and genotoxicity was tested through the formation of micronuclei (MN) and γH2AX foci, respectively. Morphology together with spectral fingerprints of nuclei were measured using enhanced dark-field microscopy (EDFM) assembled with a hyperspectral imaging (HI) module and an axial scanning fluorescence module, as well as scanning electron microscopy (SEM) coupled with energy-dispersive X-Ray spectroscopy (EDX). Cell survival was also determined in 3D SW3153 spheroids following treatment with low-LET protons with/without the IONP_DOX_ compound. IONP_DOX_ increased radiosensitivity following proton irradiation at both LETs in correlation with DNA damage expressed as MN or γH2AX. The IONP_DOX_–low-LET proton combination caused a more lethal effect compared to IONP_DOX_–high-LET protons. CHS cell biological alterations were reflected by the modifications in the hyperspectral images and spectral profiles, emphasizing new possible spectroscopic markers of cancer therapy effects. Our findings show that the proposed treatment combination has the potential to improve the management of CHS.

## 1. Introduction

Chondrosarcoma (CHS) is a radio- and chemoresistant malignant bone tumor for which surgical resection is the gold standard [1,2]. Conventional monotherapy with photon radiation and chemical agents like doxorubicin has proven ineffective on chondrosarcoma as a result of its ability to adapt [3,4,5,6]. Particle irradiation with protons and carbon ions may improve CHS treatment [5,7,8], especially due to the advantageous depth dose distribution that enables a localized high-dose deposition while minimizing the risk of toxicity to normal tissue [5,9]. To further enhance the tumor cell death, radiotherapy may be combined with nanoparticles (NPs) used as a vehicle to deliver small quantities of drugs directly to the tumor site, which increase the dose of the active substance while sparing the healthy cells [10].

In the present study, iron oxide NPs (IONPs) have been used as doxorubicin carriers [11] due to their advantages that lie in their (i) magnetic transport capacity [12,13], (ii) biocompatibility for healthy tissues as demonstrated in clinical use [14,15,16,17], and (iii) the ability to amplify radiotherapy’s efficiency by inducing the generation of reactive oxygen species in the vicinity of the NPs as well as through ROS-independent biological mechanisms [18]. The use of IONPs as radioenhancers remains scarcely studied; only few studies have highlighted the additive or synergistic effects on tumor death using IONPs in combination with conventional X-ray or particle irradiation [18,19,20,21,22,23,24,25,26]. In our previous works, we demonstrated the efficiency of dual chemotherapy–radiosensitization of photons of core–shell iron oxide NPs encapsulated in polyethylene glycol (IONP) loaded with doxorubicin (IONP_DOX_) in human cervical adenocarcinoma [11,27,28]. We also showed, for the first time, the potential of IONP_DOX_ radiosensitization towards the use of high-LET carbon ions and conventional X-ray in human CHS cells [29]. Moreover, the spectral fingerprints of cell nuclei in human CHS cells were also highlighted, for the first time, in the same study.

Taking into account these very promising findings, here, we extend our previous study [29] and evaluate the biological and radiosensitizing effects of the IONP_DOX_ nanocompound on human CHS cells, in combination with proton irradiation, since proton therapy is more available than carbon-ion therapy. This study presents novel insights into the CHS cells’ response to high- or low-energy proton irradiation in the absence or presence of IONP_DOX_ by investigating key aspects such as cell survival, DNA damage and repair, the morphology of cell organelles, spectral profiles of nuclei, and the radiosensitization effect’s dependence on proton linear energy transfer (LET). The efficiency of dual chemo-and radio-sensitization of CHS cells by IONP_DOX_ for the proton irradiation was proven, encouraging further studies with a view to finally translate it to clinics.

## 2. Results and Discussion

Considering the unique properties of proton radiation to enable the deposition of the majority of a dose at the tumor site depending on energy, this study proposes a multimodal treatment based on targeted drug delivery via IONP_DOX_ and targeted particle radiation to sensitize CHS cells in vitro. For this, the effect of drug delivery radiosensitization was investigated in both 2D and 3D cell models of CHS using low-LET protons in comparison with high-LET protons.

The uptake and retention of IONPs in 2D and 3D cultured SW1353 chondrosarcoma cells was confirmed with scanning electron microscopy (SEM) and optical microscopy, respectively. For all in vitro experiments, the cells were exposed to a selected NP concentration of 200 μg/mL and incubation times that were established in previous studies [29,30].

**Figure 1:**
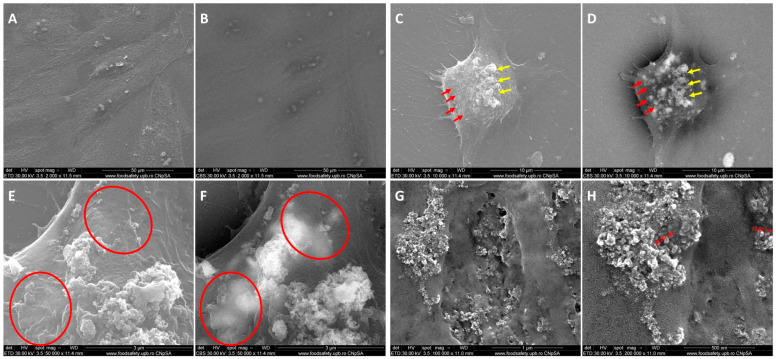
Scanning electron microscopy images of SW 1353 chondrosarcoma cells: (**A**,**B**) non-treated/control (magnification 2000×) and (**C**–**H**) treated with 200 μg/mL IONP_DOX_ (16 h); magnification: 10,000× (**C**,**D**); 50,000× (**E**,**F**); and 100,000× (**G**,**H**); the images were acquired using the secondary electron signal (**A**,**C**,**E**,**G**) and the backscattered electron signal (**B**,**D**,**F**,**H**).

SEM emphasized the internalization of IONPs in 2D SW 1353 chondrosarcoma cells by comparing the secondary electron signal (ETD) and the backscattered electron signal (CBS). In ETD (Figure 1A,C,E,G), the levels of grey represent topographic details, while in the CBS (Figure 1B,D,F,H), light greys are attributed to heavier elements (such as Fe in IONP) while darker greys are attributed to lighter elements (such as carbon in cells). Thus, by comparing the two methods of analysis, the yellow arrows in Figure 1C,D represent extracellular aggregates of IONPs, while red arrows emphasize an area where IONPs seem to be internalized in the SW 1353 cells. A similar area is highlighted by the red circles in Figure 1E,F. Figure 1G,H emphasize the interaction of IONPs with the cellular membrane; IONP internalization probably takes place through macropinocytosis mechanisms because of the specific ruffling morphology of the membrane [31]. In addition, we previously demonstrated NP localization in the perinuclear area of the SW1353 cells [29]. This will be further observed in hyperspectral images.

The internalization of IONPs in 3D SW1353 chondrosarcoma spheroids was emphasized through Prussian Blue staining (dark blue aggregates). The IONPs were observed in the spheroids, especially in their marginal area, in the marked actively metabolic cells (Figure S2A–C, Supplementary Materials).

**Figure 2:**
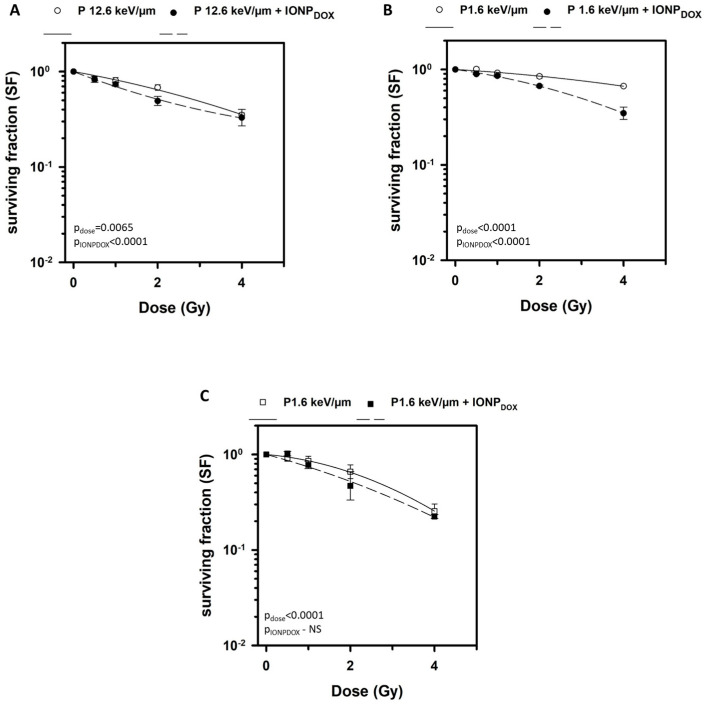
Surviving fractions of (i) 2D SW1353 chondrosarcoma cells after exposure to 200 μg/mL IONP_DOX_ for 16 h, followed by 18 MeV (**A**) or 155 MeV proton irradiation (**B**), and (ii) 3D SW1353 chondrosarcoma spheroids after exposure to 200 μg/mL IONP_DOX_ for 48 h, followed by 155 MeV proton irradiation (**C**). Data are presented as mean ± SEM (n = 3).

The potential radiosensitization effect of IONP_DOX_ was investigated by using a clonogenic survival assay. Two-dimensional chondrosarcoma cells loaded with IONP_DOX_ and then irradiated with either low- or high-energy protons showed an amplified reduction in clonogenic survival as compared to the cells irradiated with protons alone (Figure 2A,B). Indeed, the two-way ANOVA test revealed a statistically significant decrease in the survival fraction based on dose and nanoparticles (pdose = 0.0065, pNPs < 0.0001 for high LET and pdose< 0.0001, pNPs < 0.0001 for low LET). The dose-modifying-factor values expressing the effect of IONP_DOX_ on cell survival are calculated to be 0.1, 0.37 and 0.50 (Table 1). Previously, we showed that IONP_DOX_ alone induced a significant cytotoxic effect on SW1353 cells, with the survival decreasing to 0.52 ± 0.19 [29]. Both combinations, IONP_DOX_–low-LET protons and IONP_DOX_–high-LET protons, induced a sensitization effect as the DMF values are higher than 1. NP treatment followed by low-LET proton irradiation generated a much stronger sensitization of 2D chondrosarcoma cells (DMF values for all SFs around 2) than NP treatment followed by high-LET proton irradiation (DMF_SF=0.1_ = 1.098; DMF_SF=0.37_ = 1.159; DMF_SF=0.5_ = 1.398). Additionally, we analyzed the LQ models’ parameters, specifically α and β. It is known that α (Gy^−1^) results from single-track events (presents double-strand breaks of two chromosomes from a single hit) and β (Gy^−2^) arises from two-track events (indicates a double hit causing double-strand breaks of two chromosomes) [32]. Both α and β values (Table 1) were found to be increased for the combined treatment as compared to irradiation alone. Moreover, the increase in α and β values is higher for low-LET protons–IONP_DOX_ versus protons alone than for the high-LET proton–IONP_DOX_ combination versus protons alone (2.3 times and 2.2 times for α and 3.1 times and 1.3 times for β, respectively), as demonstrated by DMF values. Therefore, our results indicated the following: the high-LET protons generated less cell survival than low-LET protons; the presence of NPs intensified the cell death produced by both low and high proton irradiation alone, demonstrating a radiosensitizing potential. These results showed, for the first time, the sensitizing potential of IONP_DOX_ in CHS cells exposed to low-LET protons and confirmed our previous data obtained for the same tumor cells exposed to other types of hadrons, namely high-LET carbon ions, and also to low-LET X-ray [29]. Notably, the IONP_DOX_ caused the highest radiosensitization of CHS cells to low-LET protons as compared to carbon-ion (DMF_SF=0.1_ = 1.2 ± 0.1) and X-ray (DMF_SF=0.1_ = 1.05 ± 0.03) irradiation used in a previous study [29]. We also proved the ability of IONP_DOX_ to radiosensitize cervical adenocarcinoma HeLa cells to low-LET X-ray irradiation [28,30]. IONPs contribute to the radioenhancement effect, which causes cyto- and genotoxicity through ROS in the presence of photon or particle radiation, but also generates oxidative stress and DNA damage due to doxorubicin [18,31].

Interestingly, in the present study, the radioenhancement effect of IONP_DOX_ is higher when combined with low-LET protons as compared to high-LET protons. It is possible that the NPs induce a more lethal effect at low LET due to the interaction with the high-energy proton beam (155 MeV), which could cause secondary reactions more efficiently [32]. To date, no comprehensive explanation for this dependence on proton LET is available. Few works have exlored the dependence of proton LET on the NP radiosensitization effect and the results are contradictory. Such as, some findings indicated LET’s independence of the radiosensitization effect of AuNPs in combination with 200 MeV protons in Chinese hamster ovary CHO-K1 cells [33], while others showed a marked radiosensitization effect of GNPs with 25 keV μm⁻^1^ protons (2 MV), but not with 10 keV μm⁻^1^ protons (2 MV), in A431 cells [34].

The low-LET proton irradiation of 3D chondrosarcoma spheroids loaded with IONP_DOX_ induced a decrease in clonogenic survival, but this was not statistically significant (Figure 2C). This outcome is demonstrated by DMF values which are higher than 1 for all survival fractions—0.1, 0.37, and 0.5 (DMF_SF=0.1_ = 1.0865; DMF_SF=0.37_ = 1.121; DMF_SF=0.5_ = 1.1875)—and also by the higher α value for low-LET protons–IONP_DOX_ (0.2074 ± 0.1326) versus protons alone (0.0929 ± 0.0215). The two-way ANOVA test proved that the results were significant by means of radiation dose variation, but not in the case of IONP presence. The response of the tumor cells to NPs and radiation treatment in 3D spheroids was different compared to the 2D cell models. The radiomodulating effect in 3D chondrosarcoma spheroids was probably dependent on the nanoparticle penetration ability inside the spheroids, as previously suggested [30,35,36]. This resistant behavior might have been caused by the spheroid morphology being characterized by an extensive hypoxic area, as illustrated in Figure S2A–C (Supplementary Materials), and the lack of penetration of NPs in this area.

Given the importance of DNA stability in determining cellular propagation, we investigated the genotoxic effects of the IONP_DOX_/irradiation/combined treatment on 2D SW1353 cells through MN and γH2AX foci formation. MN reflect chromosome breakage or whole chromosome loss generated from unrepaired or mis-repaired double-strand breaks (DSBs) [37], while γH2AX is associated with DNA DSBs, being the most common surrogate marker to study DNA damage induction and the subsequent repair of the DNA lesions [38].

**Figure 3:**
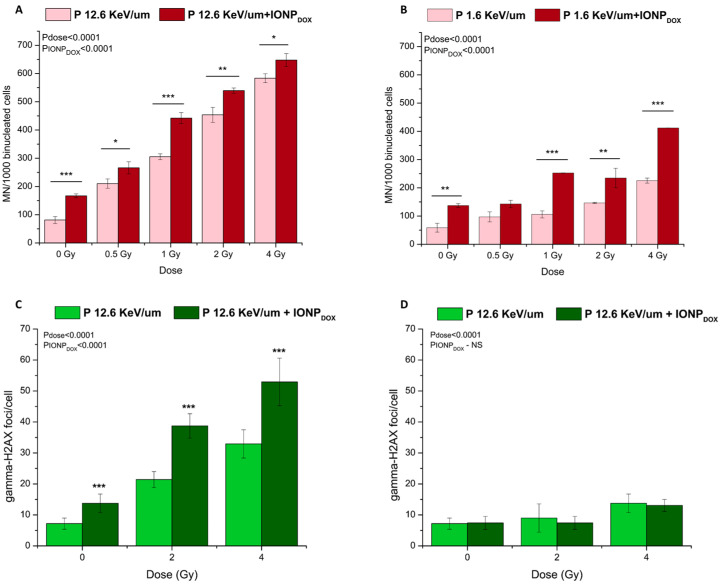
Genotoxic effects induced in 2D SW1353 chondrosarcoma cells after exposure to 200 μg/mL IONP_DOX_ for 16 h, followed by 18 MeV proton beam (**A**—micronuclei, **C**—γH2AX foci analysed at 1 h post irradiation, **D**—γH2AX foci analysed at 24 h post irradiation) or 155 MeV proton beam treatment (**B**—micronuclei); Data are presented as mean ± SEM (n = 3). * *p* < 0.5; ** *p* < 0.01; *** *p* < 0.001.

The combined treatment of IONP_DOX_ with either high-LET protons or low-LET protons significantly enhanced the induction of DNA damage, expressed as MN, as compared to the single irradiation (Figure 3A,B). As expected, high-LET protons alone or in combination with NPs caused a higher extent of DNA damage than single low-LET proton irradiation or its combination with NPs. The high level of MN in the control (higher than 50 per 1000 binucleated cells, while usually it is less than 20) and after 4 Gy irradiation (higher than 600 per 1000 MN per 1000 binucleated cells, while usually the level is around 300–400) indicates the SW1353 cell line’s high resistance to chromosomal instability. IONP_DOX_ alone induced a statistically significant MN yield as compared to the non-treated control (*p* = 0.0007) as shown in the previous paper [28]. The two-way ANOVA test confirmed that the increase in the MN number is triggered by both irradiation and the treatment with NPs (pdose < 0.0001, pNPs < 0.0001 for both cases).

**Figure 4:**
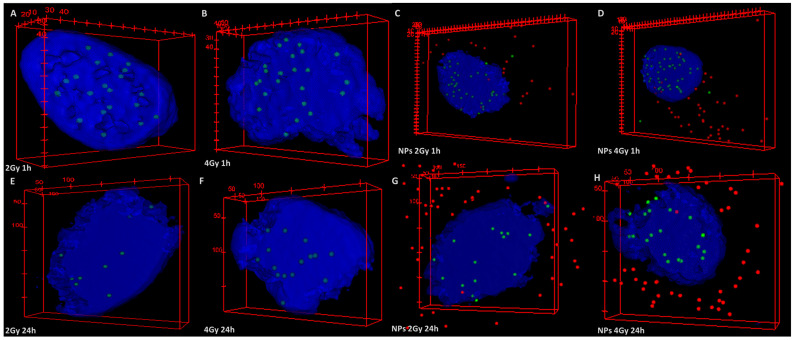
Three-dimensional reconstructions of experimental images recorded by axial scanning in fluorescence for cell nuclei: irradiated with 2 Gy (**A**,**E**) and 4 Gy (**B**,**F**) at 1 h (**A**,**B**) and 24 h (**E**,**F**) after irradiation; incubated with NPs and irradiated with 2 Gy (**C**,**D**) and 4 Gy (**F**,**H**) at 1 h (**C**,**D**) and 24 h (**G**,**H**) after irradiation. (Dimensions for points, which are γH2AX foci (green) or NPs (red), are set by the user.)

Similarly, for γH2AX foci, the following is observed (Figure 3C,D): the number of DSBs increased 1 h after treatment with NPs or high-LET proton irradiation alone (Figure 4C); the level of γH2AX foci in the control is rather high (5 per nucleus, while usually it is 4), indicating the SW1353 cell line’s resistance to chromosomal instability; the IONP_DOX_–high-LET proton combination induced a higher number of γH2AX foci at 1 h than the single irradiation as demonstrated by the two-way ANOVA test (pdose < 0.0001, pNPs < 0.0001); few residual foci remained 24 h after irradiation/irradiation–NP treatment at 4 Gy, the highest dose used in the study; this means that the repair process is almost completed within 24 h, as suggested previously in a study revealing the efficient repair of DSBs by chondrosarcoma cells after proton irradiation [39].

These outcomes could be confirmed by observing the images used for scoring the foci that resulted from using enhanced dark-field microscopy with the 3D imaging in fluorescence module (Figure 4A–H). Such an example, 3D reconstructions of the nuclei (blue) of 2D SW1353 cells irradiated with high-LET protons in the absence or presence of the nanoparticles (red) illustrate that the number of γH2AX foci (green) is higher at 1 h (Figure 4A–D) than at 24 h after irradiation (Figure 4E–H). Furthermore, the nuclei of the cells loaded with NPs and proton-irradiated exhibit a higher number of foci (Figure 4C,D,G,H) than the cells exposed to irradiation alone (Figure 4A,B,E,F). In addition, the perinuclear localization of the NPs can be observed, a result consistent with our previous data [29,40], which favors the effect of IONP-driven secondary radiation on the genetic material of the cells.

Nevertheless, all these outcomes pinpointed that the DNA damage increase contributes to the radiosensitization mechanisms of the combined treatment, IONP_DOX_ followed by proton irradiation.

Overall, our results gave evidence that the combined therapy, consisting of radiation and iron oxide NPs loaded with doxorubicin, could possibly lead to better therapeutic effects, but this has to be further demonstrated in in vivo models which are more complex than in vitro 2D and 3D models [41].

**Figure 5:**
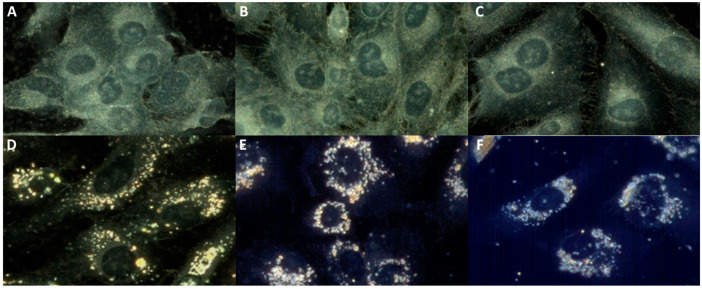
Hyperspectral images of 2D CHS cells: untreated/control (**A**); irradiated with protons (LET: 12.6 keV/µm) at 2 Gy (**B**) and 4 Gy (**C**); treated with NPs (**D**); treated with NPs and irradiated with protons (LET: 12.6 keV/µm) at 2 Gy (**E**) and 4 Gy (**F**).

The morphology of cell organelles, notably the cytoplasm and nucleus, and the spectral profile of nuclei were analyzed in order to obtain new insights into the CHS cell alteration following the NP/proton irradiation/combined treatment. Enhanced dark-field microscopy (EDFM) assembled with a hyperspectral imaging module was used for this analysis. The hyperspectral images contain details down to micro- and nanometer scales, allowing for exquisite analysis of a single cell and details inside it. The nucleus structure can be clearly seen in the HSIs (Figure 5A–C) with a well-defined area, with clean edges, darker in color, as already established in the literature [42], showing that dark-field imaging under white light illumination can be used to identify the nucleus of the cells, without the need to use any chromatographic markers.

When the cells were cultured in the presence of NPs, the images were recorded at lower exposure times to avoid CCD oversaturation due to the high intensity scattered by the IONPs (Figure 5D–F). It is observed that the NPs occupy the cytoplasm of the cells quite uniformly. Figure 5 illustrates a decrease in the cell number following irradiation with high-LET protons in the absence or presence of NPs, as also demonstrated by a clonogenic test; the internalized round-shaped NP aggregates could be observed in the perinuclear area, as observed in previous studies [11,29].

**Figure 6:**
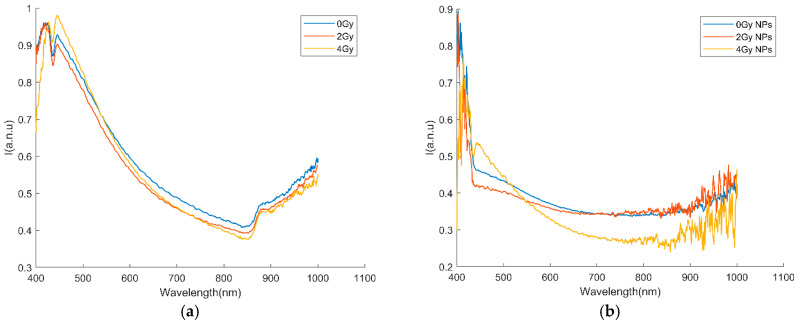
Spectral profiles as mean over all nuclei (**a**) irradiated cells without NPs; (**b**) cells irradiated and treated with NPs.

Five images from each class above were recorded and, using the region of interest (ROI) tool from the ENVI 4.8 software, the nuclei were manually segmented. The tool provides the average spectrum per each nucleus, and a home-made MATLAB script calculates the average spectra for each of the six classes above (Figure 6a,b). In the case of cells incubated without NPs, spectra with two maxima are observed; the spectrum corresponding to cells irradiated with 4 Gy reverses the intensities of the maxima, reflecting structural changes inside the nuclei. In the case of cells incubated in the presence of nanoparticles, the spectra are highly modified compared to the control, which indicates important changes in the chemical composition of the nuclei, findings that confirmed our previous results using IONP_DOX_ and carbon-ion/X-ray irradiation [29].

The differences observed between the spectral profiles of irradiated and nonirradiated cell nuclei are significant, as they enable the partitioning of HSIs into spectral sub-images. These are subsequently used to guide us to identify relevant subintervals, which serve as the basis for feature extraction and input into machine learning algorithms to compare damages induced by different types of ionizing radiation [43]. The differences between the spectral profiles become even more pronounced when we compare the irradiated cells, incubated or not with NPs (for example, compare yellow curves from Figure 6a,b).

Therefore, the spectral fingerprints of cells’ nuclei observed in HIS from unstained samples could be used to identify possible spectroscopic markers of both NPs treatment and radiotherapy, being very promising in monitoring the tumor during the treatment.

## 3. Materials and Methods

### 3.1. Cell Culture

To investigate the effect of the nanoparticle-based dual treatment, we used the chondrosarcoma cell line SW1353 (CLS Cell Lines Service GmbH, Eppelheim, Germany). The SW1353 cell line represents the most prevalent CHS subtype and is very radioresistant [3]. The cells were cultured in Dulbecco’s modified Eagle’s High-Glucose Medium (DMEM, PAN Biotech, Aidenbach, Germany) supplemented with 10% Fetal Bovine Serum (FBS, EuroClone, Via Figino, Italy), 5% L-Glutamine (Sartorius, Beit Haemek, Israel) and 1% Penicillin/Streptomycin solution (Capricorn Scientific GmbH, Ebsdorfergrund, Germany) and maintained at 37 °C in a humidified incubator with 5% CO_2_.

A standard 2D cell culture set-up was employed by using normal flat and adherent tissue culture plates (TPP, Trasadingen, Schaffhausen, Switzerland). The cells were seeded at different concentrations, depending on the set-up described below for each assay.

The 3D cell model, represented by chondrosarcoma spheroids, was obtained using the liquid-overlay technique [30,44]; 96-well plates with ultra-low adhesion and an elongated bottom for spheroid formation (Corning Incorporated, Corning, NY, USA) were used for seeding cells at concentrations between 5000 and 20,000 cells/well and kept in standard condition for 3 days to enable the formation of the spheroids. The morphology of the resulting spheroids was monitored periodically for about 14 days (T0—the first day; T14—day 14) using bright-field microscopy (Figure S1A Supplementary Materials), documenting variations in spheroid size (Figure S1B, Supplementary Materials). The optimal number of cells that led to compact spheroids was 5000 cells/well; thus, this density was selected to be used in the following experiments.

### 3.2. IONP Treatment and Internalization

Core–shell polyethylene glycol (6 kDa)-encapsulated iron oxide nanoparticles (IONPs) and nanoparticles loaded with doxorubicin (IONP_DOX_) were synthesized as previously reported [11]. The IONP_DOX_ resulting from the encapsulation of doxorubicin (1.11 wt% of doxorubicin hydrochloride) showed a mean hydrodynamic diameter of 369.1 (PDI of 0.238 and ZP of 20.9 mV) [11].

For 2D cell culture, 5 × 10^4^ cells were seeded in 12-well plates and incubated for 4 h, followed by the replacement of the culture medium with fresh medium containing IONP_DOX_ at a concentration of 200 μg/mL and incubation for another 16 h. Then, the cells were washed with PBS, and then the supernatant was replaced with nanoparticle-free fresh culture medium. Afterwards, the cells were seeded onto 10 mm round coverslips (10^4^ cells/slide) and incubated for an additional 24 h. The cells were fixed with 2.5% glutaraldehyde for 1 h and dehydrated using ethanol solutions of 70%, 90% and 100%, for 30 min each. Then, the cells were incubated using ethanol/hexamethyldisilasane solutions of 50:50%, 25:75%, and 0:100%, respectively, for 6 min each.

The uptake and retention of the IONP nanocompound in the 3D SW1353 cells was investigated by optical microscopy, as follows: the spheroids were collected after seeding and resuspended in fresh culture medium with nanoparticles (at a concentration of 200 µg/mL), then incubated for another 48 h in the presence of nanoparticles, washed with PBS, fixed using 4% PFA in PBS, incubated in 30% sucrose in PBS, mounted in cryogel, and placed at −80 °C overnight. The samples obtained were cryosectioned at a depth of 6 μm and collected on microscopic slides. The samples were stained using Mayer hematoxylin for 10 min at room temperature; the nanoparticles were colored with Prussian blue solution (potassium hexacyanoferrate trihydrate, Merck-Sigma Aldrich, Darmstadt, Germany), then mounted with glycerol.

### 3.3. Cell Irradiation

The cells were irradiated with protons using two facilities: (1) the TR-19 Cyclotron research facility from IFIN-HH (Magurele, Romania), generating a beam of 18 MeV protons with an LET of 12.6 keV/μm, and the (2) Phasotron clinical facility from JINR, Dubna, Russia, generating a beam of 155 MeV with an LET of 1.6 keV/μm. The LET of 12.6 keV/μm was obtained from the native beam of an 18 MeV cyclotron by placing a methyl polymethacrylate (PMMA) filter between the sample and the beam exit area. The LET of 1.6 keV/μm was obtained from the native beam of 155 MeV protons by using PMMA blocks placed directly in front of the samples, which corresponds to the real conditions of patient irradiation as much as possible. The total thickness of the PMMA blocks was 145 mm of water equivalent. Thus, the samples were approximately in the center of the modified Bragg peak. In our study, we have termed the lowest LET value for protons as “low-LET” and the highest as “high-LET”, with the purpose of separately comparing the two LET values.

For irradiation with a proton beam line of 18 MeV, the 2D cell cultures were obtained by seeding 5 × 10^4^ cells/well in 12-well plates (TPP Techno Plastic Products AG, Trasadingen, Switzerland) and treating them as described above. Before exposure, plates were sealed with breathable sealing tape (Corning, NY, USA).

For irradiation with the proton beam line of 155 MeV, the 2D cell cultures were obtained by seeding 5 × 10^6^ cell in 25 cm^2^ flasks (TPP Techno Plastic Products AG, Trasadingen, Switzerland) and treating them as described above. Then, the cells were washed to remove nanoparticle excess, detached from the flask, transferred to 1.5 mL Eppendorf tubes, and exposed to the proton beam.

For irradiation with the proton beam line of 155 MeV protons, 3D spheroids were treated with nanoparticles as described above, washed, collected from the plates, transferred to 1.5 mL Eppendorf tubes, and exposed to irradiation. Three-dimensional spheroids were irradiated only with high-energy protons and not with the low-energy protons, which do not provide enough penetration depth in tissues, given the energy of the particles.

Cells with and without IONP_DOX_ were exposed to 0, 0.5, 1, 2 and 4 Gy.

### 3.4. Colony Formation Assay

For both 2D and 3D models, the colony formation assay was performed immediately after irradiation. The cells were detached in a single cell suspension using trypsin (0.25%/EDTA 0.02% solution; Capricorn Scientific GmbH, Ebsdorfergrund, Germany) or Accutase (STEMCELL Technologies, Vancouver, BC, Canada) and seeded at 800 cells/well and 6333 cells/well, respectively, in 6-well plates.

The plates were incubated for 14 days under standard conditions of temperature and humidity, followed by fixation, coloring with crystal violet (Sigma-Aldrich Chemie GmbH, Burlington, MA, USA), and counting the colonies containing at least 50 cells. The plating efficiency (PE), which represents the percentage of seeded cells that survive to form colonies under control conditions, and the survival fraction (SF), which is the fraction of cells that survive or die due to the treatment (e.g., radiation, drug, etc.) that one is testing, are calculated according to the following formulas:

(1) $$PE=\frac{number\;of\;counted\;colonies}{number\;of\;seeded\;cells}\times 100$$

(2) $$SF=\frac{number\;of\;counted\;colonies}{number\;of\;seeded\;cells\;\times \;PE/100}$$

The survival fraction (SF) was fitted with the linear–quadratic model (ln(SF) = −(αD + βD2)), where D is the given dose and α and β are constants calculated through the fitting procedure using the non-linear regression tool of SigmaPlot 15 (Systat Software GmbH, Erkrath, Germany) with an extension provided by Heidelberg University [45]. The IONP_DOX_ radiosensitization potential was evaluated by normalization to the control (IONP_DOX,_ nonirradiated cells). This fitting process, facilitated by specialized software tools for radiobiological data analysis, generates the values for the α and β constants. The dose modification factor (DMF) was calculated to show the variation in the absorbed dose due to the presence of NPs, defined as DMF = Dc_ontrol_/D_NPs_, where D is the dose producing a specified biological effect.

### 3.5. Cytokinesis-Block Micronucleus Assay

For each experimental condition, the 2D chondrosarcoma cells were prepared as described in the colony formation assay, and then 10^4^ cells were seeded on 10 mm coverslips and incubated for approximatively 1 h in order to attach, followed by the addition of Cytochalasin B (Sigma-Aldrich Chemie GmbH, Burlington, MA, USA) at a concentration of 3 μg/mL in culture medium and incubation for 20 h. Then, the cells were fixed using an acetic acid–methanol solution (1:9) and stained using acridine orange (Sigma-Aldrich Chemie GmbH, Burlington, MA, United States) at a concentration of 10 µg/mL in PBS. The micronuclei were scored in 1000 binucleated cells according to recommended criteria [46] by using an epifluorescence microscope, the Olympus BX-51 (Olympus, Hamburg, Germany).

### 3.6. Gamma-H2AX Immunofluorescence Analysis

SW 1353 chondrosarcoma cell samples were prepared and treated as described in the micronucleus assay at a density of 10^4^. After 1 h and 24 h of incubation following irradiation, cells were fixed with paraformaldehyde 3.7% in PBS for 10 min at room temperature. Following 1% bovine serum albumin blocking and permeabilization with Triton X, the cells were incubated with the primary antibody (anti-gamma H2Ax 1:200 in PBS) for 1 h at room temperature. The cells were rinsed multiple times with PBS before incubation with the secondary antibody (anti-mouse-FITC 1:200 in PBS) for 1 h at room temperature. Hoechst counterstaining of nuclei was performed (10 µg/mL, 10 min at room temperature). The slides were mounted with glycerol at room temperature prior to Cytoviva 3D microscopy examinations using the 3D imaging in fluorescence module as described below (Section 3.7).

### 3.7. Three-Dimensional and Hyperspectral Image Acquisition and Processing

Cell images were obtained using enhanced dark-field microscopy with two modules: hyperspectral imaging in white light and 3D imaging in fluorescence. We used the Cytoviva commercial system (CytoViva, Inc., Auburn, AL, USA); the transmission configuration, with a patented cardioid shape condenser, was illuminated through optical fibers. The radiation scattered on the details of the sample is transmitted to the microscope objective (60×, 1.25NA). Both the condenser and the objective are oil-immersed.

For the hyperspectral module, the lighting source is a FiberLite DC-950 (white light, 150 W quartz halogen aluminum reflector, Dolan Jenner Industries, Boxborough, MA, USA), and the image recording system consists of a spectrophotometer (ImSpectrum V10E, Specim, Oulu, Finland) with a transmission diffraction grating inserted between the objective and a hyperspectral camera (Pixelfly 1392 × 1040-pixel resolution, 6.45 × 6.45 µm pixel size, 7.3 to 13.5 fps, 5 µs-60 s exposure time range, 62% quantum efficiency).

For the module with axial scanning in fluorescence for 3D imaging, the lighting source is a mercury vapor lamp (Lumen200, Prior Scientific Instruments Ltd., Cambridge, UK), and for the acquisition of images, the system is equipped with cooled EXiBlue monochrome CCD (QIMAGING Corporation, Surrey, BC, Canada, 1392 × 1040 pixels, 15 fps at maximum resolution, 6.45 × 6.45 µm pixel size). The fluorescent filters used in this study were for DAPI and FITC staining.

The hyperspectral images are recorded by transversal scanning, and the fluorescence images are recorded by axial scanning. Both modules use the motorized stage (NanoScanZ, Prior Scientific Instruments Ltd., Cambridge, UK, 10 nm step size, 114 × 75 mm travel range).

Hyperspectral images are recorded on unlabeled samples, located in their culture medium. We used the standard acquisition procedure, and we set the exposure time from 0.001 s to 0.6 s for cells incubated with NPs to avoid CCD saturation but to ensure sufficient spectral data. The field of view (maximum 696 × 696 pixels) allows the recording of large colonies of attached cells. This configuration allows high spatial (107.5 nm) and spectral (1.28 nm) resolution (covering the entire visible and near-infrared spectrum (400 nm to 1000 nm). For the data analysis, the hyperspectral signal was output via the dedicated ENVI software; lamp correction was performed, and the spectral profiles were obtained (at each single pixel or on areas employing the “region of interest” (ROI) tool).

To record images by axial scanning in fluorescence, the samples are fluorescently labeled as described in the previous section. Few parameters need to be set: exposure time (1s for images recorded with fluorescent filters and around 500 ms for images recorded without fluorescent filters), the number of slices (NZ = 61 for our samples) which will be recorded in each scan, and the distance between them (Δz = 100 nm in our experiments). Slices are parallel to the glass slide on which the cells are grown. Without changing anything in terms of the position of the sample on the microscope stage (focalization distance, NZ and Δz), we axially scanned the sample three times in our case: once with the F1 filter (DAPI, emission centered at 461 nm) inserted before the condenser to obtain the image of the nucleus, the second with the F2 filter (FITC emission centered at 530 nm) inserted before the condenser to observe Gamma H2AX foci inside the nuclei, and the third without filters to obtain the experimental images of light radiation scattered by NPs (in the case of samples incubated with NPs). In this way, three sets of slices are registered: F1-Zstacks for nucleus, F2-Zstacks for Gamma H2AX foci, and WL-Zstacks for nanoparticles. For images recorded with fluorescence filters, the exposure time is set to avoid CCD saturation, but keeping the edges of the nuclei continuous. For the images recorded without filters, the exposure times are much shorter, because the intensity of the radiation scattered by the NPs is very high.

The processing of these images to obtain 3D representations is performed using special plugins in ImageJ, provided by the manufacturer. The procedure contains steps to obtain a point spread function and deconvolution for the images recorded with the DAPI filter, in order to remove the unfocused details from the image of the nucleus. For the images recorded with FITC and without filters, we ran the “Just locate nanoparticles” plugin. Assembling all three types of images into one 3D image is carried out according to a procedure specific to our laboratory [47].

### 3.8. Statistical Analysis

The results were presented as the mean ± SEM and resulted from a minimum of three experiments, with three replicates for each condition in each experiment. Student’s *t*-test or two-way ANOVA (GraphPad Prism 8.2, La Jolla, CA, USA) were used for statistical analysis. A *p*-value of less than 0.05 (* *p* < 0.05, ** *p* < 0.01, *** *p* < 0.001) indicates statistical significance for the results.

## Figures and Tables

**Table 1:** The calculated DMF values and radiobiological parameters of the linear quadratic model for 2D and 3D SW1353 cells treated with low-LET or high- LET protons in the presence or absence of IONP_DOX._

Cell Model	Treatment	LET keV/μm	α (Gy^−1^)	β (Gy^−2^)	R^2^	DMF SF0.1	DMF SF0.37	DMF SF0.5
2D	18 MeV Protons	12.6	0.1801 ± 0.0496	0.0199 ± 0.0138	0.9812	-	-	-
	18 MeV Protons + IONP_DOX_	12.6	0.3881 ± 0.0307	0.0270 ± 0.0086	0.9941	1.098 ± 0.272	1.159 ± 0.228	1.398 ± 0.220
	155 MeV Protons	1.6	0.0599 ± 0.0185	0.0103 ± 0.0052	0.984	-	-	-
	155 MeV Protons + IONP_DOX_	1.6	0.1379 ±0.0169	0.0315 ±0.0047	0.998	2.011± 0.118	2.028 ± 0.119	2.041 ± 0.127
3D	155 MeV Protons	1.6	0.0929 ± 0.0215	0.0620± 0.0060	0.9981	-	-	-
	155 MeV Protons + IONP_DOX_	1.6	0.2074 ± 0.1326	0.0436 ± 0.0436	0.9507	1.087 ± 0.162	1.121 ± 0.392	1.1875 ± 0.681

## Data Availability

The original contributions presented in the study are included in the article/Supplementary Material, further inquiries can be directed to the corresponding author.

## Supplementary Materials

The following supporting information can be downloaded at: https://www.mdpi.com/article/10.3390/ijms252111481/s1.

## References

[1] Laitinen M.K., Parry M.C., Le Nail L.R., Wigley C.H., Stevenson J.D., Jeys L.M. (2019). Locally Recurrent Chondrosarcoma of the Pelvis and Limbs Can Only Be Controlled by Wide Local Excision. Bone Jt. J..

[2] Zając A.E., Kopeć S., Szostakowski B., Spałek M.J., Fiedorowicz M., Bylina E., Filipowicz P., Szumera-Ciećkiewicz A., Tysarowski A., Czarnecka A.M. (2021). Chondrosarcoma-from Molecular Pathology to Novel Therapies. Cancers.

[3] de Jong Y., Ingola M., Briaire-de Bruijn I.H., Kruisselbrink A.B., Venneker S., Palubeckaite I., Heijs B.P.A.M., Cleton-Jansen A.-M., Haas R.L.M., Bovée J.V.M.G. (2019). Radiotherapy Resistance in Chondrosarcoma Cells; a Possible Correlation with Alterations in Cell Cycle Related Genes. Clin. Sarcoma Res..

[4] Catanzano A.A., Kerr D.L., Lazarides A.L., Dial B.L., Lane W.O., Blazer D.G., Larrier N.A., Kirsch D.G., Brigman B.E., Eward W.C. (2019). Revisiting the Role of Radiation Therapy in Chondrosarcoma: A National Cancer Database Study. Sarcoma.

[5] Gilbert A., Tudor M., Montanari J., Commenchail K., Savu D.I., Lesueur P., Chevalier F. (2023). Chondrosarcoma Resistance to Radiation Therapy: Origins and Potential Therapeutic Solutions. Cancers.

[6] Tlemsani C., Larousserie F., De Percin S., Audard V., Hadjadj D., Chen J., Biau D., Anract P., Terris B., Goldwasser F. (2023). Biology and Management of High-Grade Chondrosarcoma: An Update on Targets and Treatment Options. Int. J. Mol. Sci..

[7] Guan X., Gao J., Hu J., Hu W., Yang J., Qiu X., Hu C., Kong L., Lu J.J. (2019). The Preliminary Results of Proton and Carbon Ion Therapy for Chordoma and Chondrosarcoma of the Skull Base and Cervical Spine. Radiat. Oncol..

[8] Riva G., Cavallo I., Gandini S., Ingargiola R., Pecorilla M., Imparato S., Rossi E., Mirandola A., Ciocca M., Orlandi E. (2021). Particle Radiotherapy for Skull Base Chondrosarcoma: A Clinical Series from Italian National Center for Oncological Hadrontherapy. Cancers.

[9] Grau C., Durante M., Georg D., Langendijk J.A., Weber D.C. (2020). Particle Therapy in Europe. Mol. Oncol..

[10] Bilynsky C., Millot N., Papa A.L. (2022). Radiation Nanosensitizers in Cancer Therapy—From Preclinical Discoveries to the Outcomes of Early Clinical Trials. Bioeng. Transl. Med..

[11] Popescu R.C., Savu D., Dorobantu I., Vasile B.S., Hosser H., Boldeiu A., Temelie M., Straticiuc M., Iancu D.A., Andronescu E. (2020). Efficient Uptake and Retention of Iron Oxide-Based Nanoparticles in HeLa Cells Leads to an Effective Intracellular Delivery of Doxorubicin. Sci. Rep..

[12] Gong X., Wang F., Huang Y., Lin X., Chen C., Wang F., Yang L. (2018). Magnetic-Targeting of Polyethylenimine-Wrapped Iron Oxide Nanoparticle Labeled Chondrocytes in a Rabbit Articular Cartilage Defect Model. RSC Adv..

[13] Liu X., Zhang H., Zhang T., Wang Y., Jiao W., Lu X., Gao X., Xie M., Shan Q., Wen N. (2022). Magnetic Nanomaterials-Mediated Cancer Diagnosis and Therapy. PBioE.

[14] Amag Pharmaceuticals https://covispharma.com/index.php/products/.

[15] MagForce https://magforce.de/.

[16] Fortuin A.S., Meijer H., Thompson L.C., Alfred Witjes J., Barentsz J.O. (2013). Ferumoxtran-10 Ultrasmall Superparamagnetic Iron Oxide-Enhanced Diffusion-Weighted Imaging Magnetic Resonance Imaging for Detection of Metastases in Normal-Sized Lymph Nodes in Patients with Bladder and Prostate Cancer: Do We Enter the Era after Extended Pelvic Lymph Node Dissection?. Eur. Urol..

[17] Zamecnik P., Israel B., Feuerstein J., Nagarajah J., Gotthardt M., Barentsz J.O., Hambrock T. (2022). Ferumoxtran-10-Enhanced 3-T Magnetic Resonance Angiography of Pelvic Arteries: Initial Experience. Eur. Urol. Focus.

[18] Ternad I., Penninckx S., Lecomte V., Vangijzegem T., Conrard L., Lucas S., Heuskin A.C., Michiels C., Muller R.N., Stanicki D. (2023). Advances in the Mechanistic Understanding of Iron Oxide Nanoparticles’ Radiosensitizing Properties. Nanomater.

[19] Ibáñez-Moragues M., Fernández-Barahona I., Santacruz R., Oteo M., Luján-Rodríguez V.M., Muñoz-Hernando M., Magro N., Lagares J.I., Romero E., España S. (2023). Zinc-Doped Iron Oxide Nanoparticles as a Proton-Activatable Agent for Dose Range Verification in Proton Therapy. Molecules.

[20] Seo S.-J., Jeon J.-K., Jeong E.-J., Chang W.-S., Choi G.-H., Kim J. (2013). Enhancement of Tumor Regression by Coulomb Nanoradiator Effect in Proton Treatment of Iron-Oxide Nanoparticle-Loaded Orthotopic Rat Glioma Model: Implication of Novel Particle Induced Radiation Therapy. J. Cancer Ther..

[21] Khoei S., Mahdavi S.R., Fakhimikabir H., Shakeri-Zadeh A., Hashemian A. (2014). The Role of Iron Oxide Nanoparticles in the Radiosensitization of Human Prostate Carcinoma Cell Line DU145 at Megavoltage Radiation Energies. Int. J. Radiat. Biol..

[22] Kirakli E.K., Takan G., Hoca S., Müftüler F.Z.B., Kılçar A.Y., Kamer S.A. (2018). Superparamagnetic Iron Oxide Nanoparticle (SPION) Mediated in Vitro Radiosensitization at Megavoltage Radiation Energies. J. Radioanal. Nucl. Chem..

[23] Klein S., Sommer A., Distel L.V.R., Hazemann J.L., Kröner W., Neuhuber W., Müller P., Proux O., Kryschi C. (2014). Superparamagnetic Iron Oxide Nanoparticles as Novel X-Ray Enhancer for Low-Dose Radiation Therapy. J. Phys. Chem. B.

[24] Hauser A.K., Mitov M.I., Daley E.F., McGarry R.C., Anderson K.W., Hilt J.Z. (2016). Targeted Iron Oxide Nanoparticles for the Enhancement of Radiation Therapy. Biomaterials.

[25] Russell E., Dunne V., Russell B., Mohamud H., Ghita M., McMahon S.J., Butterworth K.T., Schettino G., McGarry C.K., Prise K.M. (2021). Impact of Superparamagnetic Iron Oxide Nanoparticles on in Vitro and in Vivo Radiosensitisation of Cancer Cells. Radiat. Oncol..

[26] Shetake N.G., Kumar A., Pandey B.N. (2019). Iron-Oxide Nanoparticles Target Intracellular HSP90 to Induce Tumor Radio-Sensitization. Biochim. Biophys. Acta. Gen. Subj..

[27] Popescu R.C., Savu D.I., Bierbaum M., Grbenicek A., Schneider F., Hosser H., Vasile B.Ș., Andronescu E., Wenz F., Giordano F.A. (2021). Intracellular Delivery of Doxorubicin by Iron Oxide-Based Nano-Constructs Increases Clonogenic Inactivation of Ionizing Radiation in Hela Cells. Int. J. Mol. Sci..

[28] Popescu R.C., Kopatz V., Andronescu E., Savu D.I., Doerr W. (2023). Nanoparticle-Mediated Drug Delivery of Doxorubicin Induces a Differentiated Clonogenic Inactivation in 3D Tumor Spheroids In Vitro. Int. J. Mol. Sci..

[29] Tudor M., Popescu R.C., Negoita R.D., Gilbert A., Ilisanu M.A., Temelie M., Dinischiotu A., Chevalier F., Mihailescu M., Savu D.I. (2023). In Vitro Hyperspectral Biomarkers of Human Chondrosarcoma Cells in Nanoparticle-Mediated Radiosensitization Using Carbon Ions. Sci. Rep..

[30] Popescu R.C., Straticiuc M., Mustăciosu C., Temelie M., Trușcă R., Vasile B.Ș., Boldeiu A., Mirea D., Andrei R.F., Cenușă C. (2020). Enhanced Internalization of Nanoparticles Following Ionizing Radiation Leads to Mitotic Catastrophe in MG-63 Human Osteosarcoma Cells. Int. J. Mol. Sci..

[31] Aroui S., Brahim S., Waard M.D., Kenani A. (2010). Cytotoxicity, intracellular distribution and uptake of doxorubicin and doxorubicin coupled to cell-penetrating peptides in different cell lines: A comparative study. Biochem. Biophys. Res. Commun..

[32] Kuncic Z., Lacombe S. (2018). Nanoparticle radio-enhancement: Principles, progress and application to cancer treatment. Phys. Med. Biol..

[33] Cunningham C., de Kock M., Engelbrecht M., Miles X., Slabbert J., Vandevoorde C. (2021). Radiosensitization Effect of Gold Nanoparticles in Proton Therapy. Front. Public Health.

[34] Li S., Penninckx S., Karmani L., Heuskin A.C., Watillon K., Marega R., Zola J., Corvaglia V., Genard G., Gallez B. (2016). LET-dependent radiosensitization effects of gold nanoparticles for proton irradiation. Nanotechnology.

[35] Tchoryk A., Taresco V., Argent R.H., Ashford M., Gellert P.R., Stolnik S., Grabowska A., Garnett M.C. (2019). Penetration and Uptake of Nanoparticles in 3D Tumor Spheroids. Bioconjug. Chem..

[36] Sánchez G.J., Maury P., Stefancikova L., Campion O., Laurent G., Chateau A., Hoch F.B., Boschetti F., Denat F., Pinel S. (2019). Fluorescent Radiosensitizing Gold Nanoparticles. Int. J. Mol. Sci..

[37] Heaven C.J., Wanstall H.C., Henthorn N.T., Warmenhoven J.W., Ingram S.P., Chadwick A.L., Santina E., Honeychurch J., Schmidt C.K., Kirkby K.J. (2022). The Suitability of Micronuclei as Markers of Relative Biological Effect. Mutagenesis.

[38] Sharma A., Singh K., Almasan A. (2012). Histone H2AX Phosphorylation: A Marker for DNA Damage. Methods Mol. Biol..

[39] Lohberger B., Glänzer D., Eck N., Kerschbaum-Gruber S., Mara E., Deycmar S., Madl T., Kashofer K., Georg P., Leithner A. (2021). Activation of Efficient DNA Repair Mechanisms after Photon and Proton Irradiation of Human Chondrosarcoma Cells. Sci. Rep..

[40] Petcov T.E., Straticiuc M., Iancu D., Mirea D.A., Trușcă R., Mereuță P.E., Savu D.I., Mogoșanu G.D., Mogoantă L., Popescu R.C. (2024). Unveiling Nanoparticles: Recent Approaches in Studying the Internalization Pattern of Iron Oxide Nanoparticles in Mono- and Multicellular Biological Structures. J. Funct. Biomater..

[41] Holub A.R., Huo A., Patel K., Thakore V., Chhibber P., Erogbogbo F. (2020). Assessing Advantages and Drawbacks of Rapidly Generated Ultra-Large 3D Breast Cancer Spheroids: Studies with Chemotherapeutics and Nanoparticles. Int. J. Mol. Sci..

[42] Pinto R.J.B., Bispo D., Vilela C., Botas A.M.P., Ferreira R.A.S., Menezes A.C., Campos F., Oliveira H., Abreu M.H., Santos S.A.O. (2020). One-Minute Synthesis of Size-Controlled Fucoidan-Gold Nanosystems: Antitumoral Activity and Dark Field Imaging. Materials.

[43] Negoita R.D., Ilisanu M.A., Irimescu I.N., Popescu R.C., Tudor M., Mihailescu M., Scarlat E.N., Pleava A.M., Dinischiotu A., Savu D. (2024). Specific spectral sub-images for machine learning evaluation of optical differences between carbon ion and X ray radiation effects. Heliyon.

[44] Carlsson J., Yuhas J.M. (1984). Liquid-Overlay Culture of Cellular Spheroids. Recent Results Cancer Res..

[45] Burger N., Biswas A., Barzan D., Kirchner A., Hosser H., Hausmann M., Hildenbrand G., Herskind C., Wenz F., Veldwijk M.R. (2014). A Method for the Efficient Cellular Uptake and Retention of Small Modified Gold Nanoparticles for the Radiosensitization of Cells. Nanomedicine Nanotechnology. Biol. Med..

[46] Fenech M. (2007). Cytokinesis-Block Micronucleus Cytome Assay. Nat. Protoc..

[47] Mihailescu M., Miclea L.C., Pleava A.M., Tarba N., Scarlat E.N., Negoita R.D., Moisescu M.G., Savopol T. (2023). Method for Nanoparticles Uptake Evaluation Based on Double Labeled Fluorescent Cells Scanned in Enhanced Darkfield Microscopy. Biomed. Opt. Express.

