# Supplementary material for: Enhancing Proton Radiosensitivity of Chondrosarcoma Using Nanoparticle-Based Drug Delivery Approaches: A Comparative Study of High- and Low-Energy Protons
Source: Int J Mol Sci. 2024 Oct 25;25(21):11481. doi: 10.3390/ijms252111481 (PMC11546389; doi:10.3390/ijms252111481)
Supplement: Supplementary file 1 [file ijms-25-11481-s001.zip › ijms-3236680-supplementary.pdf]

International Journal of *Molecular Sciences*

MDPI

*Supplementary Material for the article*

# Enhancing Proton Radiosensitivity of Chondrosarcoma Using Nanoparticle-Based Drug Delivery Approaches: A Comparative Study of High- and Low-Energy Protons

**Mihaela Tudor [1,2,†], Roxana Cristina Popescu [1,3,†], Ionela N. Irimescu [4], Ann Rzyanina [5], Nicolae Tarba [6], Anca Dinischiotu [2], Liviu Craciun [7], Tiberiu Relu Esanu [7], Eugeniu Vasile [8], Andrei Theodor Hotnog [9], Mihai Radu [1], Gennady Mytsin [5], Mona Mihailescu [10,11] and Diana Iulia Savu [1,*]**

[1] Department of Life and Environmental Physics, Horia Hulubei National Institute of Physics and Nuclear Engineering, Reactorului 30, P.O. Box MG-6, 077125 Magurele, Romania; mihaela.tudotr@nipne.ro (M.T.); roxana.popescu@nipne.ro (R.C.P.); mradu@nipne.ro (M.R.)
[2] Faculty of Biology, University of Bucharest, Splaiul Independentei 91-95, 050095 Bucharest, Romania; anca.dinischiotu@bio.unibuc.ro
[3] Department of Bioengineering and Biotechnology, Faculty of Medical Engineering, National University for Science and Technology Politehnica of Bucharest, Gheorghe Polizu Street, 1-7, 011061 Bucharest, Romania
[4] Applied Sciences Doctoral School, National University for Science and Technology Politehnica of Bucharest, 060042 Bucharest, Romania; ionela.irimescu@stud.fsa.upb.ro
[5] Laboratory of Nuclear Problems, Joint Institute for Nuclear Research, 6 Joliot-Curie Street, 141980 Dubna, Moscow Region, Russia; rzjanina@mail.ru (A.R.); mytsin@mail.ru (G.M.)
[6] Doctoral School of Computer Sciences, National University for Science and Technology Politehnica of Bucharest, 060042 Bucharest, Romania; nicolae.tarba@upb.ro
[7] Radiopharmaceutical Research Centre, Horia Hulubei National Institute of Physics and Nuclear Engineering, 077125 Magurele, Romania; cliviu@nipne.ro (L.C.); tiberiu.esanu@nipne.ro (T.R.E.)
[8] Faculty of Applied Physics, National University for Science and Technology Politehnica of Bucharest, 060042 Bucharest, Romania; eugeniu.vasile@upb.ro
[9] Applied Nuclear Physics Department, Horia Hulubei National Institute of Physics and Nuclear Engineering, Reactorului 30, P.O. Box MG-6, 077125 Magurele, Romania; andrei.hotnog@nipne.ro
[10] Holographic Imaging and Processing Laboratory, Physics Department, National University for Science and Technology Politehnica of Bucharest, 060042 Bucharest, Romania; mona.mihailescu@upb.ro
[11] Centre for Research in Fundamental Sciences Applied in Engineering, National University for Science and Technology Politehnica of Bucharest, 060042 Bucharest, Romania
† These authors contributed equally to this work.
* Correspondence: dsavu@nipne.ro

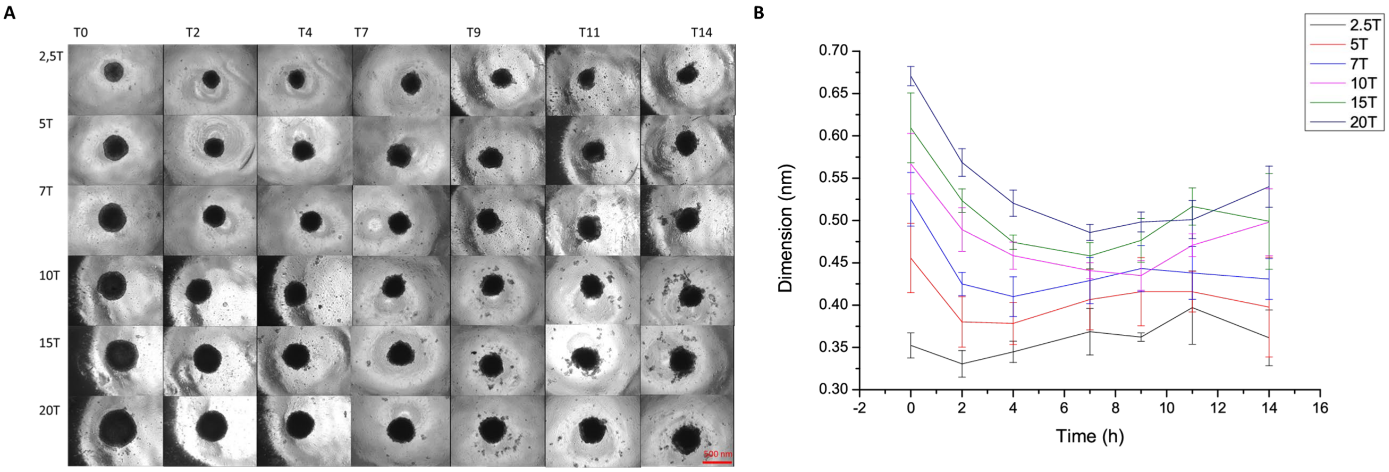


**Figure S1.** Three-dimensional model (spheroids) of SW1353 chondrosarcoma cells: (**A**) optical microscopy images (5x magnification), and (**B**) spheroid dimensions at different initial cell concentrations measured over time.

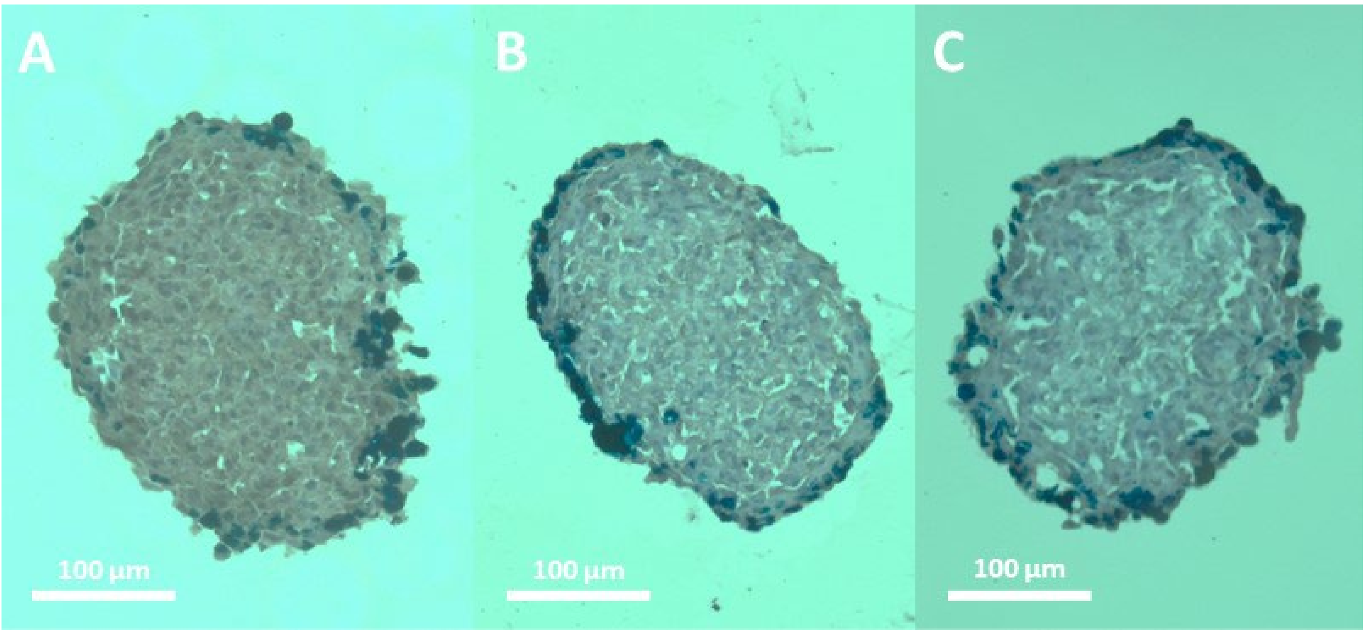


**Figure S2.** Bright-field microscopy images of IONPDOX (administered concentration of 200 µg/mL) internalization in 3D SW1353 cell model after 48h of incubation (**A**–**C**); cells- violet (Mayer Hematoxylin), IONP- blue (Prussian Blue).
